# Influential factors of school bullying among junior high school students: an analysis based on hierarchical linear models

**DOI:** 10.3389/fpsyg.2025.1664252

**Published:** 2026-01-05

**Authors:** Yingying Wang, Xingyu Li, Nianhao Song, Changhao Jiang, Pei Liu

**Affiliations:** 1 School of Kinesiology and Health, Capital University of Physical Education and Sports, Beijing, China; 2 The Center of Neuroscience and Sports, Capital University of Physical Education and Sports, Beijing, China; 3 School of Physical Education, Capital University of Physical Education and Sports, Beijing, China; 4 Affiliated Competitive Sports School, Capital University of Physical Education and Sports, Beijing, China

**Keywords:** junior high school students, school bullying, hierarchical linear models, China education panel survey data, school type

## Abstract

**Introduction:**

Exploring the factors and internal mechanisms influencing school bullying among junior high school students is crucial for preventing and controlling its occurrence. Such research provides a reference for supporting students’ physical and mental health development and promoting the high-quality advancement of education.

**Methods:**

This study based on data from the China Education Panel Survey (2014–2015), this study employed hierarchical linear modeling to examine the individual- and school-level factors associated with bullying in junior high schools. The analysis focused on identifying predictors at both levels and investigating their interactive mechanisms.

**Results:**

At the individual level, gender, academic achievement, health status, disciplinary behaviors, and parental discipline were significant predictors of school bullying. At the school level, school type significantly predicted bullying prevalence. Furthermore, moderation analysis indicated that school type moderated the relationship between parental discipline and bullying. Specifically, the negative association between parental discipline and bullying was stronger in private schools compared to public schools.

**Discussion:**

This study reveals both the direct effects and the interactive mechanisms of factors influencing school bullying among junior high school students. The findings offer valuable insights for developing practical and effective prevention and intervention strategies, particularly by considering the moderating role of school type.

## 1 Introduction

School bullying is defined as the repeated and prolonged engagement in harmful behaviors by an individual student or a group of students targeting a specific person or group within a school setting (Olweus, 2013). Bullying at school has been extensively examined for its long-term consequences on individuals’ mental and physical health, and several studies have shown that it can be harmful (Hughes et al., 2025). According to Hymel and Swearer, bullying in school settings involves more than just physical assaults; it also includes verbal abuse, social marginalization, and the distinct features of cyberbullying (Hymel and Swearer, 2015; Romera et al., 2022). Technological advancements have been identified as a contributing factor in the increasing prevalence of cyberbullying (Jones et al., 2013). According to the World Health Organization, one in three children worldwide has experienced bullying by peers (Singham et al., 2017). Bullying in educational institutions is a serious problem in China as well. According to research, between 2 and 66% of mainland Chinese adolescents, or the bulk of the adolescent population, experience bullying at school (Han et al., 2017). School bullying demonstrated to be closely associated with a number of factors affecting adolescents, including, but not limited to, academic performance, cognitive development, physical health, mental well-being, and social adaptation (Arseneault et al., 2010; Lenzi et al., 2015; O’Brennan and Furlong, 2010). Research indicates that, in comparison with adolescents who have not been subjected to bullying, those who experience elevated levels of bullying demonstrate diminished social adaptation and academic performance (Arslan, 2022). Furthermore, these individuals are more prone to the development of feelings of inferiority, anxiety, depression, somatization, and behavioral issues. In severe cases, this may result in suicidal behavior (Alikasifoglu et al., 2007; Arslan, 2022; Moore et al., 2017), significantly impacting adolescents’ growth and development.

Individual factors in adolescents are closely associated with the occurrence of school bullying. Among these, gender is a significant factor influencing bullying behavior. The majority of studies indicate that boys experience bullying at a higher frequency than girls (Carrera Fernandez et al., 2013; Feijóo and Rodríguez-Fernández, 2021). Furthermore, there is heterogeneity in the types of bullying experienced by the two groups, boys are more likely to be subjected to physical bullying, while girls are more likely to experience verbal or relational bullying (Carrera Fernandez et al., 2013; García-Fernández et al., 2022). Body size is also an important factor influencing school bullying, with obese children being more likely to experience verbal and relational bullying than children of normal weight (Gong et al., 2020; Morales et al., 2019). Additionally, family economic conditions may also have a certain influence on the occurrence of school bullying. Children from economically advantaged families with higher social status are less likely to become victims of bullying (Elgar et al., 2013). However, parental emotional support can reduce the risk of children from economically disadvantaged families becoming victims of bullying (Jiang and Liang, 2025). Furthermore, parenting styles are also associated with children’s bullying behaviors, harsh parental discipline positively predicted school bullying among Chinese adolescents (Fan et al., 2023; Kokkinos and Panayiotou, 2007). Therefore, to gain a clearer understanding of the risks associated with school bullying among adolescents, it is essential to consider individual-level factors.

Schools are the primary settings where bullying occurs, with most incidents taking place in classrooms, playgrounds, hallways, and restrooms (Francis et al., 2022). Bowes argues that school size is a significant predictor of the occurrence of bullying, as larger schools may underestimate the risk of children being bullied due to teachers’ inability to fully understand each student’s situation (Bowes et al., 2009). School atmosphere also has is also correlated with the occurrence of bullying. A positive school atmosphere reduces the likelihood of bullying, while a negative school atmosphere weakens students’ emotional connection to the school, increasing the likelihood of bullying (Fink et al., 2018). Additionally, the type of school is an important factor influencing bullying. School type is generally determined by its sponsoring institution. Public schools are funded by government departments and tend to have more comprehensive resources and management systems, while non-public schools are funded by individuals or groups and have relatively limited recognition. Research indicates that approximately 7% of the probability of bullying occurring is attributed to differences between schools (Kärnä et al., 2011). Compared to public schools, non-public schools have a higher probability of language and relational bullying occurring (Harris et al., 2019).

However, existing research still has certain limitations. First, most existing studies explore the concepts, phenomena, harms, and related legal systems of school bullying from a theoretical perspective, while relatively few empirical studies. Second, existing studies are mostly based on data from a single region or a few regions, with small sample sizes, resulting in limited representativeness of the findings. Finally, some studies only analyze the causes of school bullying from a single perspective, with few studies considering multiple perspectives comprehensively. Based on this, this study aims to utilize the China Education Panel Survey (CEPS) data, which features rigorous design, a large sample size, and broad regional coverage, to employ Hierarchical Linear Models (HLM) to explore the influencing factors of school bullying perpetration among junior high school students from both the individual and school levels, and to provide valuable references for the prevention and intervention strategies of bullying issues.

## 2 Methods

### 2.1 Data sources

**Figure 1:**
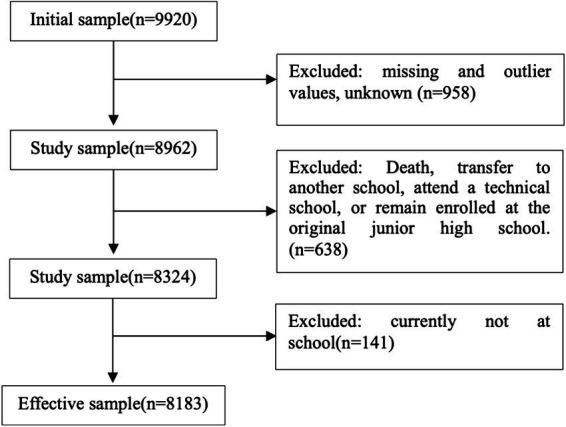
Data screening flowchart.

The study utilized data from the CEPS (2014–2015), which is China’s first national, longitudinal, and representative large-scale tracking survey targeting secondary school students. The survey employed the Probability Proportional to Size Sampling (PPS), randomly selecting 28 counties, 112 schools, and 438 classes of students nationwide for inclusion in the sample. The initial sample size was 9,920. Missing and outlier values for core variables were removed as needed for the study, resulting in a final effective sample size of 8,183 (Figure 1).

### 2.2 Variable description

This study examined school bullying behavior as the dependent variable, measured through four CEPS questionnaire items assessing verbal abuse, swearing, quarreling, physical fighting, and bullying of weaker classmates (Nansel et al., 2001; Olweus, 2013), with response coding detailed in Table 1.

**Table 1:** Definition of the influencing variables of school bullying among junior high school students.

Variable type	Variable name	The corresponding item in CEPS	Description of the variable
Explanatory variables	School bullying		
	School bullying	Have you ever scolded or sworn in the past year? Have you quarreled in the past year? Have you fought in the past year? Have you bullied weak classmates in the past year?	Never = 1, Occasionally = 2, Sometimes = 3, Often = 4, Always = 5
Explanatory variables	Individual level		
	Gender	What is your gender?	Female = 0, Male = 1
	Only child	Are you an only child?	No = 0, Yes = 1
	Academic performance	Do you find it difficult to learn math now Do you find it difficult to learn Chinese now Do you find it difficult to learn English now?	Very hard = 1, a little hard = 2, not very hard = 3, not at all = 4
	Health status	How healthy are you now?	Poor = 1, Not very good = 2, Average = 3, Fairly good = 4, Good = 5
	Family economic conditions	What do you think of your family’s current economic situation?	Very poor = 1, Relatively poor = 2, Average = 3, Relatively wealthy = 4, Very wealthy = 5
	Disciplinary behavior	Have you skipped class, skipped, or skipped school in the past year? Have you plagiarized assignments or cheated on exams in the past year? Have you plagiarized smoking or drinking alcohol in the past year? In the past year, have you ever used an Internet café or a game arcade?	Never = 1, Occasionally = 2, Sometimes = 3, Often = 4, Always = 5
	Parental discipline	Your parents are strict with you in homework and exams? Your parents are strict with you in school? Your parents are strict with who you make friends with? Your parents are strict about how you dress? Your parents are strict about your online time? Your parents are strict about how much time you spend watching TV?	Regardless = 1, A litter strict = 2, Very strict = 3
	School level		
	School type	The type of your school belongs to	Public schools were designated as the reference category; Two dummy variables were created, private school, coded as 1 for private schools and 0 otherwise; other school, coded as 1 for private schools and 0 otherwise.

This study uses the factors influencing school bullying as independent variables, including individual-level and school-level variables. Individual-level variables included gender, only child, academic performance, health status, family economic conditions, disciplinary behaviors, and parental discipline. School-level variables included school type, this variable was operationalized as a categorical distinction based on formal governance and funding sources. Public schools were defined as institutions funded and regulated by local or national educational authorities, while non-public schools included private, independent, or parochial institutions funded primarily through tuition, donations, or private grants. No inherent evaluative judgments (e.g., “good” or “bad”) were attached to this classification; it served solely to capture structural differences between school systems. The definitions of each variable and the coding of questionnaire options are shown in Table 1.

### 2.3 Building a model

The HLM method was originally proposed by Raudenbush and Chan (1993) for the analysis of non-independent data characterized by a multi-level nested structure. In this study, individual students were nested within schools, thus the research model includes two levels: students and schools. In the context of model estimation, the Maximum Likelihood Estimation (MLE) was utilized for the purpose of comparing log-likelihood values and evaluating the overall goodness of fit of the model. The feasibility of utilizing a multilevel model was assessed by calculating intra-class correlation coefficients (ICC), while cross-level interactions were employed to examine the moderating effect of community factors on individual factors. The specific model is as follows:

The initial tier of HLM was the individual level. It is posited that the coefficients at the individual level are capable of explaining the differences in bullying behaviors attributable to student characteristics. In this model, Y_ij_ denotes the value of bullying behavior for the i-th student in the j-th school, while β_0k_ represents the value of bullying behavior for students in the k-th school when all variables are set to 0. X_kij_ signifies the value of the i-th student in the k-th school across the j individual variables, and β_kj_ denotes the estimated coefficient. Finally, ε_ik_ represents the random error. The specific model is illustrated in Equation 1.


$$Y_{\mathrm{ij}}=\beta_{0k}+\sum_{j=1}^{n}\beta_{\mathrm{kj}}X_{\mathrm{kij}}+\varepsilon_{\mathrm{ik}}$$ (1)

The second level of HLM was the school level. This level provided an explanation of the impact of different school-level characteristics on bullying among junior high school students. In this model, r₀ represents of the constant term at the school level; q is the number of school-level variables; Z_qk_ is the value of the q variables at the kth school; r_₀q_ is the estimated coefficient for Z_qk_; and μ_0k_ is the random error at the school level, indicating the deviation between the mean of bullying behavior among students at the j-th school and the overall mean of bullying behavior among all students. The specific models are illustrated in Equations 2 and 3.


$$\beta_{0k}=r_{0}+\sum_{q=1}^{m}r_{0q}Z_{\mathrm{qk}}+\mu_{0k}$$ (2)

$$\beta_{\mathrm{kj}}=r_{j}$$ (3)

Finally, substitute the school-level equation into the individual-level equation to obtain the complete HLM model, where μ_0k_ and ε_ik_ are both assumed to follow a normal distribution and are independent of each other. The specific form is shown in Equation 4.


$$Y_{\mathrm{ij}}=r_{0}+\sum_{q=1}^{m}r_{0q}Z_{\mathrm{qk}}+\sum_{j=1}^{n}r_{j}X_{\mathrm{kij}}+\mu_{0k}+\varepsilon_{\mathrm{ik}}$$ (4)

### 2.4 Data analysis

The present study utilized SPSS 26.0 to perform data analysis. Firstly, a descriptive statistical analysis was conducted on the selected variables. Secondly, Pearson correlation analysis was employed to explore the correlations among the core variables, thereby providing preliminary evidence for subsequent research. A multilevel regression model incorporating individual and community factors was constructed, based on the HLM analysis method. The moderation effect analysis was conducted using the PROCESS macro (Model 1), systematically examining the independent effects and interaction effects of factors at different levels on school bullying among junior high school students.

## 3 Results

### 3.1 The results of descriptive statistics and correlation

Table 2 presents the descriptive statistics and correlation analysis results for each core variable. The results indicate that there is a significant positive correlation between junior high school students’ school bullying and disciplinary violations (r = 0.553, *p* < 0.001) and school type (r = 0.054, *p* < 0.001), and a significant negative correlation between school bullying and parental discipline (r = −0.173, *p* < 0.001).

**Table 2:** Descriptive statistics and correlation analysis results of variables.

Variable	M ± SD	Bullying	Disciplinary behaviors	Parental discipline
Bullying	1.634 ± 0.581			
Disciplinary behaviors	1.219 ± 0.435	0.553***		
Parental discipline	2.272 ± 0.411	−0.173***	−0.214***	
School type	1.130 ± 0.530	0.054***	0.118***	−0.003

*p < 0.05, **p < 0.01, ***p < 0.001.

### 3.2 Results of HLM

As shown in the results of Model 1 (Table 3), the between-group variance is 0.022 (*p* < 0.001), indicating that there are significant differences in school-level factors in school bullying behavior. The ICC was calculated as 0.0.022/(0.022 + 0.315) = 0.065, indicating that approximately 0.065 of the variation in bullying behavior among junior high school students is attributable to school-level factors. Based on the criterion that “the ICC is greater than 0.059”(Cohen, 1988), it can be determined that HLM should be used for model construction.

**Table 3:** Results of model 1.

Random effects	Variance	Standard error	*p*-value	Log-likelihood (ML)
Bullying (ε_ik_)	0.022	0.004	0.000	13974.423
Individual level (μ_0k_)	0.315	0.005		

Model 2 was primarily used to examine the independent effects of individual-level variables on school bullying. As shown in the results of Table 4, gender, academic performance, health status, and parental discipline all have a significant negative predictive effect on school bullying. For each additional unit of parental discipline, the incidence of school bullying decreases by 0.066 units. Being an only child and disciplinary violations both positively predicted the occurrence of school bullying. Specifically, for each additional unit of disciplinary violations, the incidence rate of school bullying increased by 0.682 units. However, family economic conditions did not have a significant predictive effect on school bullying.

**Table 4:** Results for each model.

Analysis level	Variable	Model 2	Model 3	Model 4
Individual level	Gender	−0.094^***^ (0.011)	−0.094^***^ (0.010)	−0.094^***^ (0.011)
	Only child	0.037^**^ (0.012)	0.034 (0.012)	0.033^**^ (0.012)
	Academic performance	−0.048^***^ (0.009)	−0.050^***^ (0.010)	−0.050^***^ (0.009)
	Health status	−0.023^***^ (0.006)	−0.024^**^ (0.006)	−0.024^***^ (0.006)
	Disciplinary behavior	0.682^***^ (0.013)	0.680^***^ (0.013)	0.748^***^ (0.028)
	Family economic conditions	0.015 (0.010)	0.016 (0.010)	0.017 (0.010)
	Parental discipline	−0.066^***^ (0.013)	−0.064^***^ (0.013)	−0.153^***^ (0.032)
School level	School type		−0.025 (0.019)	−0.118 (0.072)
Interactions	Disciplinary behavior * School type			−0.058^**^ (0.021)
	Parental discipline * School type			0.079^**^ (0.025)
	Constant terms	1.139^***^ (0.054)	1.179^***^ (0.058)	1.280^***^ (0.098)
	Log-likelihood value	10936.799	10693.906	10672.320

***p < 0.001, **p < 0.01, *p < 0.05, only variables that have statistically significant interactions are listed.

**Figure 2:**
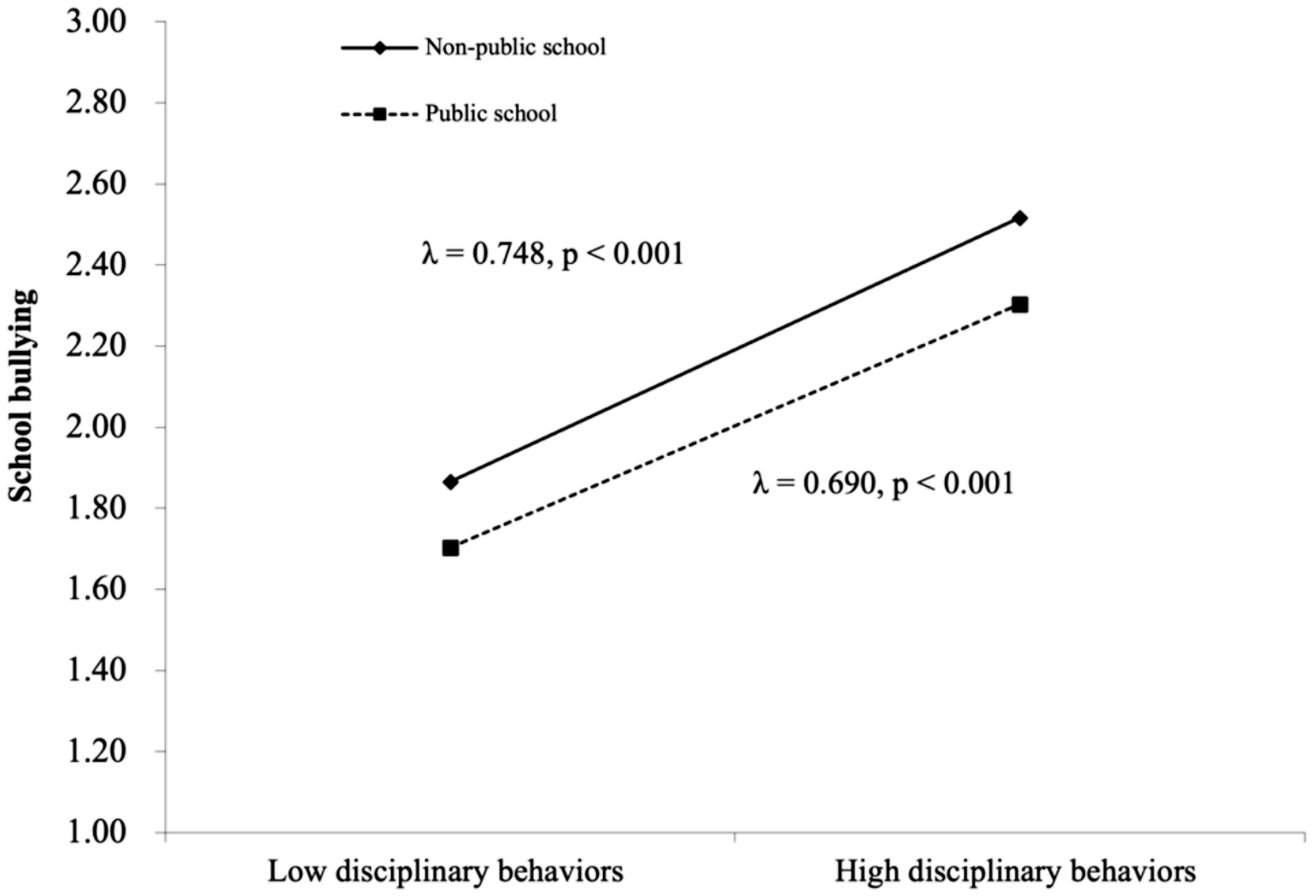
Moderating effect of school type between disciplinary behaviors and school bullying.

**Figure 3:**
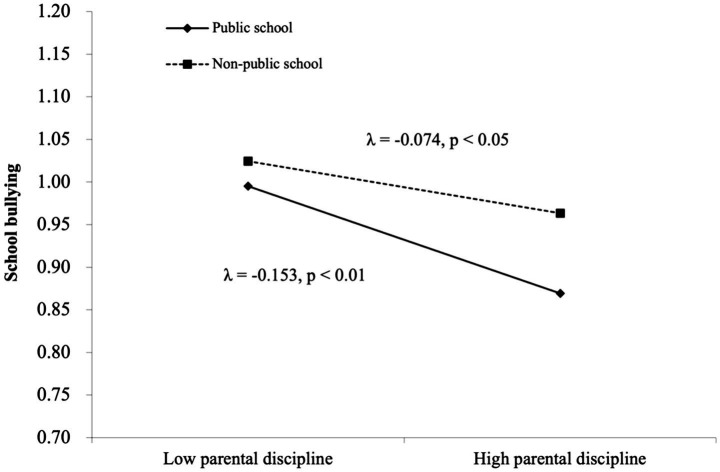
Moderating effect of school type between parental discipline and school bullying.

Model 3 built upon Model 2 by incorporating school-level variables, testing only the independent effects of school-level variables on school bullying without interaction terms. At the school level, the results indicated that school type does not significantly predict school bullying (*p* > 0.05). Model 4 was the full model, which not only analyzes the independent effects of various influencing factors on school bullying but also examines cross-level interaction effects. The results indicated that there is a significant interaction between school-level variables and individual-level variables related to school bullying among junior high school students. First, the relationship between disciplinary behavior and school bullying differed across public and non-public schools. Specifically, the association was stronger in non-public school compared to public schools. Second, *school type significantly moderated the relationship between parental discipline and school bullying*, meaning that *the negative association between parental discipline and bullying was stronger in non-public schools compared to public schools.* Following the approach of Preacher et al. (2006), the simple slope method was used, with M ± 1SD as the standard, to represent high and low values of the moderation effect. The moderation effects were shown in Figures 2, 3.

## 4 Discussion

This study based on CEPS (2014–2015) data and utilized the HLM to investigate the direct and interactive effects of variables at both individual and school levels on the occurrence of bullying behaviors among junior high school students, aiming to explain the multifaceted factors contributing to school bullying.

### 4.1 Individual-level variables and bullying among junior school students

Research has found that gender significantly influences school bullying among junior high school students, with boys being considerably more likely to be involved in school bullying than girls. This finding is consistent with Evans et al. (2013). This difference may be related to the socialization differences in gender roles during adolescence, as boys are more inclined to express themselves through overt behaviors and are thus more susceptible to physical bullying such as hitting and slapping (Nansel et al., 2001). Research has found that non-only children are more likely to be bullied. The dispersion of family resources may result in reduced emotional support and material resources for individuals. Neglect in emotional, physical, and familial support affects the development of children’s self-perception (Rees, 2008), making them more prone to involvement in school bullying incidents. Studies have also revealed a significant negative correlation between academic performance and school bullying. Within the context of the Chinese education system, where academic achievement serves as a key evaluation criterion, students with poor academic performance may face risks of social exclusion, discrimination, and ridicule, thereby increasing the likelihood of being bullied (Fu et al., 2013; Shaheen et al., 2018). This finding indicates cultural differences from studies conducted in some Western countries (Jennlfer et al., 2011). In addition, health status has a significant negative effect on school bullying. Students with better health are less likely to become targets of bullying. Conversely, children who are physically weak, timid, or lack self-protection abilities are more vulnerable to being bullied (Smokowski and Kopasz, 2005). Research also shows that disciplinary behaviors significantly affect the occurrence of school bullying. This may be due to the contrast between junior high school students’ pursuit of novelty and excitement and the monotony of school life; some students skip classes or stay away from school in search of new experiences (Zottis et al., 2014), while others resort to school bullying as a means of self-expression, seeking identity recognition, or emotional release.

In addition, research has found that family economic income significantly and positively predicts school bullying. Students from families with better economic income tend to have parents with higher educational attainment, who are more willing to invest time and effort in their children’s education and development, thereby promoting children’s cognitive development (Cave et al., 2022). Furthermore, families with higher economic conditions establish protective mechanisms for their children through increased educational investment and access to quality schools, shielding them from bullying issues. Research also indicates that parental discipline can influence the occurrence of school bullying. Reasonable parental discipline and high levels of support can mitigate the negative impact of low family economic income on children bullying behaviors (Liu et al., 2025), fostering healthy personality development, stronger psychological resilience, and the ability to face life’s challenges with positivity and optimism, thus reducing the likelihood of being bullied (Garaigordobil and Machimbarrena, 2017).

### 4.2 School-level variables and bullying among junior high school students

Research has found that school type also significantly influences the occurrence of bullying among junior high school students, consistent with the findings of Harris’s study (Harris et al., 2019). Specifically, public schools have the lowest incidence of bullying, and as school type shifts from public to private, the probability of bullying increases (Kelly et al., 2016). In China, most public schools receive government financial support, have high-quality faculty, well-equipped facilities, and strict disciplinary management, thereby better meeting the expectations of parents and society. In contrast, private schools for migrant workers’ children often have limited educational resources, inadequate campus facilities and environments, weak faculty, and high staff turnover, which increases school bullying incidents.

### 4.3 The moderating effect of school-level variables on bullying in junior schools

Research has found a significant interaction effect between disciplinary behaviors and school type, indicating that school type moderates the relationship between disciplinary behaviors and school bullying. Specifically, the key public schools typically have a long history of operation, are often located in central urban areas, and enjoy high-quality educational resources and well-established management systems, with relatively strict disciplinary standards. Students attending such schools tend to come from families with higher economic income, and these characteristics effectively reduce the likelihood of school bullying (Due et al., 2009; Fu et al., 2013).

The study further indicates that school type significantly moderates the relationship between parental discipline and school bullying. Public schools, with their professional teaching staff and comprehensive support systems, can effectively mitigate the negative effects of inadequate parenting discipline. Ecological Systems Theory posits that interaction among nested environments shape child development. Parental discipline (microsystem) and school type (mesosystem) are interdependent and modulate the protective effect of discipline against bullying. Notably, the buffering effect of parental discipline is more pronounced in public schools with high-quality teachers and standardised management. This is because behavioral norms are aligned between home and school: when parents set clear boundaries, the public school’s unified anti-bullying protocols and structured classroom management reinforce these norms. This consistency eliminates ambiguity in behavioral expectations, thereby reducing the risk of bullying. Additionally, due to their high teaching standards, teachers can promptly identify students’ psychological needs through daily observations and provide necessary emotional support, thereby reducing the risk of such students being subjected to school bullying (Zeng et al., 2023).

### 4.4 Limitations

This study provides a novel perspective on the factors influencing school bullying among junior high school students based on HLM, but it still has some limitations. Firstly, the phenomenon of school bullying is influenced by a multitude of factors, and the indicators selected in this study are limited. Future research could expand the dimensions of indicator selection for a more comprehensive exploration of the phenomenon. Secondly, the data utilized in this study were collected during the period 2014–2015. It is recommended that future research utilize data from recent years in order to achieve a more precise understanding of the current influencing factors. Third, this study is notable for its exclusive focus on the direct effects and interaction effects of two-level factors on school bullying among junior high school students. Future research could incorporate the family level to explore the roles of three-level factors, thereby further deepening the study. Fourth, our data analysis did not account for the influence of regional hierarchical factors (such as socioeconomic characteristics or cultural norms). Future research focusing on cross-regional comparisons would benefit significantly from adopting a multilevel model framework. This approach would allow researchers to more directly examine how macro-level contextual factors shape relationship patterns at the individual level.

## 5 Conclusion

This study systematically examined the relevant factors influencing school bullying among junior high school students. At the individual level, gender, academic performance, health status, disciplinary behaviors, and parental discipline all had significant influence school bullying among junior high school students. At the school level, school type had a significant influences school bullying behavior among junior high school students. Furthermore, the school type moderates the relationship between student disciplinary behaviors and bullying among junior high school students, and also moderated the relationship between parental discipline and bullying among junior high school students. This study provided valuable insights into the mechanisms underlying bullying among junior high school students and offered important implications for effective prevention and control of bullying in schools.

## Data Availability

The original contributions presented in the study are included in the article/supplementary material, further inquiries can be directed to the corresponding author/s.
